# The prognostic role of the low and very low baseline LDL-C level in outcomes of patients with cardiac revascularization; comparative registry-based cohort design

**DOI:** 10.1186/s13019-023-02333-y

**Published:** 2023-07-28

**Authors:** Malihe Rezaee, Aida Fallahzadeh, Ali Sheikhy, Ali Ajam, Saeed Sadeghian, Mina Pashang Bsc, Mahmoud Shirzad, Soheil Mansourian, Jamshid Bagheri, Kaveh Hosseini

**Affiliations:** 1 Department of Pharmacology, School of Medicine, Shahid Beheshti University of Medical Sciences, Tehran, Iran; 2 Non-Communicable Disease Research Center, Endocrinology and Metabolism Population Sciences Institute, Tehran University of Medical Sciences, Tehran, Iran; 3 Cardiovascular Diseases Research Institute, Tehran Heart Center, Tehran University of Medical Sciences, North Karegar Ave, P.O. Box: 1411713138, Tehran, Iran; 4 Department of Cardiac Surgery, Tehran University of Medical Sciences, Tehran, Iran

**Keywords:** Coronary artery bypass grafting, Coronary artery disease, Baseline low-density lipoprotein cholesterol, Low-density lipoprotein cholesterol, All-cause mortality, Outcomes

## Abstract

**Background:**

Although low-density lipoprotein-cholesterol (LDL-C) level is considered one of the main prognostic factors in patients with coronary artery bypass grafting (CABG), the question about “the lower the better” is still unanswered. We aimed to evaluate and compare the outcomes of patients with CABG and low or very low baseline LDL-C, regardless of statin usage.

**Methods:**

In this registry-based cohort study, 10,218 patients with low/very low (70–100 and ≤ 70 mg/dL) baseline LDL-C who underwent isolated and the first-time CABG without known previous history of cardio-cerebrovascular events, were included and compared. The median follow-up was 73.33 (72.15–74.51) months. Primary outcomes were all-cause mortality and major adverse cardio-cerebrovascular events (MACCE) (consisted of all-cause mortality, acute coronary syndrome, stroke or transient ischemic attack, and the need for repeat revascularization [percutaneous coronary intervention or redo-CABG]). Cox regression analyses before and after the propensity score matching (PSM) model were applied to evaluate and compare outcomes.

**Results:**

The mean age of the study population was 66.17 ± 9.98 years old and 2506 (24.5%) were women. Diabetes mellitus and a history of cigarette smoking were significantly higher in the very low LDL group (P-value ≤ 0.001). In Cox regression analyses before applying PSM model, both all-cause mortality (14.2% vs. 11.9%, P-value = 0.004 and MACCE (26.0% vs. 23.6%, P-value = 0.006) were significantly higher in the very low LDL group compared to low LDL. However, these results were no longer significant after applying the PSM model (all-cause mortality HR: 1.115 [95% CI: 0.986–1.262], P = 0.083 and MACCE HR: 1.077 [95%CI: 0.984–1.177], P = 0.095). The sensitivity analysis to remove the statin effect demonstrated that very low LDL-C level was correlated to higher risk of all-cause mortality in both unmatched and PSM analyses.

**Conclusion:**

Very low serum LDL-C levels (≤ 70 mg/dl) could increase long-term all-cause mortality and cardiovascular events in patients who have undergone isolated CABG.

**Supplementary Information:**

The online version contains supplementary material available at 10.1186/s13019-023-02333-y.

## Introduction

Coronary artery disease (CAD) is still the leading cause of morbidity and mortality worldwide which imposes great costs on healthcare systems [1]. There are many modifiable and non-modifiable risk factors for CAD [2]. Several perioperative risk factors have been recognized that predict survival after coronary artery bypass grafting (CABG) and identifying these predictors is crucial in the clinical management of patients [3, 4].

Increased serum LDL-C is widely regarded as one of the major independent risk factors for the development of CAD and is associated with a worse prognosis [5]. Several studies have demonstrated that a strategy of aggressive LDL-C lowering (approximately ≤ 100 mg/dl and ≤ 70 mg/dl in high-risk patients) with high-dose statin therapy before and after CABG is associated with lower postoperative morbidity and mortality compared to moderate LDL-C lowering [6–10]. Therefore, previous studies suggested that very low LDL-C levels have a necessarily better cardiovascular outcome. However, further investigations shown that low baseline LDL-C levels could paradoxically be associated with worse outcomes in some patients with CAD; this is known as the cholesterol paradox [11]. In this line, a recent study indicated that not only lower baseline LDL-C levels were not associated with improved outcomes, but also were linked to a poor prognosis [12].

The benefits of lowering LDL-C after CABG are well-recognized, with available evidence recommend achieving LDL-C levels below 55 mg/dL as ideal in patients who underwent CABG [13]. Nevertheless, the prognostic value of very low baseline LDL-C at the time of admission has not been properly determined in patients who underwent isolated CABG. In this study, we will be evaluating and comparing the prognostic value of low (70 mg/dl < LDL-C ≤ 100 mg/dl) and very low (LDL-C ≤ 70 mg/dl) perioperative LDL-C in these patients.

## Methods

### Study design

**Fig. 1:**
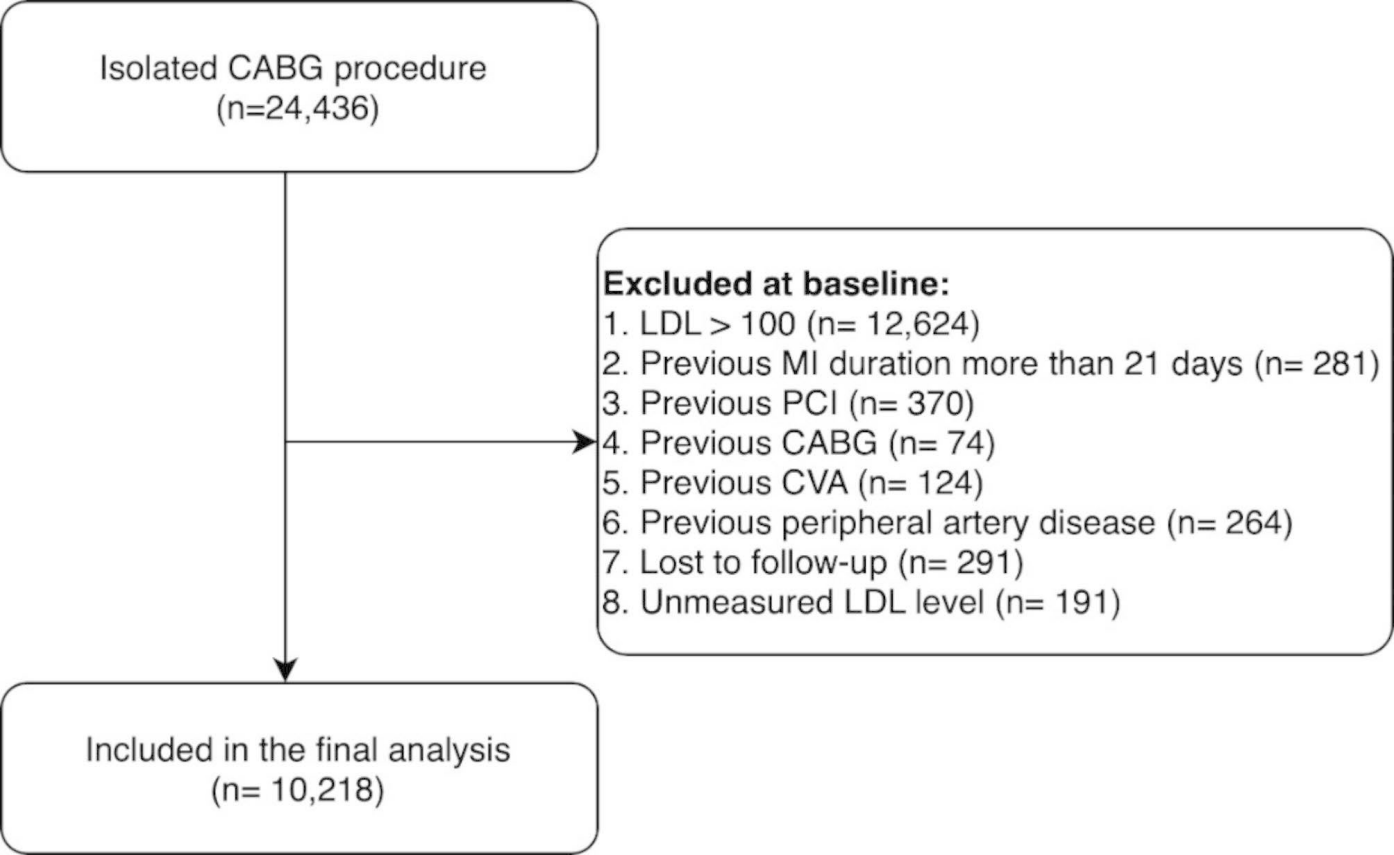
inclusion and exclusion criteria

This is a registry-based prospective study conducted in Tehran Heart Center (THC) clinical registry, which includes patients with CAD who underwent isolated CABG between 2005 and 2015. The study was approved by Tehran Heart Center ethical board (IR-THC-13,799). This study didn’t meet the criteria for informed consent whereas the patient names were kept anonymous except for the corresponding author and database chief, thus “informed consent waiver” was obtained from the Tehran Heart Center ethical board. Involving human data was in accordance with the guidelines of the Declaration of Helsinki. We assessed all patients who underwent isolated CABG, and patients with inadequate data were excluded from the current study. Among 24,328 patients who underwent isolated CABG in our database, we only included patients with LDL-C levels of under 100 mg/dl in the study, moreover, patients with a lack of sufficient data were excluded from the final analysis, ultimately, 10,218 patients were analyzed in the final model (Fig. 1).

### Follow-up protocol

The patients were followed at 4 or 6 and 12 months after surgery and yearly thereafter through direct visits. Patients who were unable to attend in-person clinic visits were followed up through telephone interviews by healthcare specialists, who asked them to send their laboratory documents and managed their medications. Also, all patients were managed, based on the last updated version of ESC guideline, after CABG and during follow-up. The details of the protocol of this registry were previously described [14]. The patients’ demographic characteristics, CAD risk factors (i.e., diabetes mellitus (DM), hypertension (HTN), dyslipidemia (DLP), family history of CAD, cigarette smoking (CS), opium consumption, and obesity), laboratory results (hemoglobin and creatinine), history of previous disease, ejection fraction, number of grafts, and occurrence of MACCE were recorded.

DM was defined as fasting plasma glucose ≥ 126 mg/dL and/or random plasma glucose ≥ 200 mg/dL and/or hemoglobin A1c (HbA1c) ≥ 6.5% [15] and/or treatment with either oral hypoglycemic agents or insulin. HTN was defined as a minimum systolic blood pressure of 140 mm Hg or a minimum diastolic blood pressure of 90 mm Hg or a history of receiving antihypertensive therapy [16]. DLP was defined as the presence of a minimum total cholesterol level of 240 mg/dL, a minimum triglyceride level of 200 mg/dL, a high-density lipoprotein cholesterol (HDL-C) level of less than 40 mg/dL in men, and less than 50 mg/dL in women, a minimum LDL-C level of 160 mg/dL, or a history of prescribed lipid-lowering medications based on the National Cholesterol Education Program (NCEP) Adult Treatment Plan (ATP) III [17, 18] A family history of CAD was defined as having a first-degree relative with a history of CAD including acute myocardial infarction or documented CAD (through invasive coronary angiography or computed tomography coronary angiography). CS and opium consumption were determined based on the patient’s self-reported status. A current smoker was defined as an individual who has ever smoked more than 100 cigarettes and who currently smokes [19]. Opium consumption was defined as the current consumption of opium either smoking opium or drinking opium dissolved in tea.

### Study endpoints

The primary endpoints of this study were all-cause mortality and occurrence of MACCE (composite of all-cause mortality, acute coronary syndrome, stroke or transient ischemic attack, and the need for repeat revascularization [percutaneous coronary intervention or redo-CABG]). Secondary outcomes included non-fatal cardiovascular events (CVEs), in-hospital mortality, length of ICU admission, and intubation time.

### Statistical analysis

Mean with standard deviation (SD) and median with 25th and 75th percentiles (interquartile range [IQR] boundaries) were used to present normally and skewed distributed continuous variables, respectively. The normality of the variables was assessed using histogram charts in addition to central tendency and dispersion measures. Comparison between “low LDL-C” and “very-low LDL-C” groups was done using student’s t-test for normally distributed and Mann-Whitney U-test for skewed distributed variables. Categorical variables were expressed as frequency and percentage which were compared between the two abovementioned groups using the chi-squared test. The adjusted and unadjusted effects of very low LDL-C levels on all-cause mortality and MACCE were obtained using Cox’s proportional hazards (PH) model and reported as hazards ratio (HR) with 95% confidence intervals. The Proportional hazard assumption was tested through a graphical assessment based on the scaled Schoenfeld residuals for each final model’s variable. The proportional hazard assumption was met for all mentioned variables. To minimize the effects of reverse causality, a sensitivity analysis was conducted on statin-user patients. The one-to-one nearest neighborhood propensity score matching (PSM) technique (considering caliper as 0.01) without replacement was conducted to balance the baseline characteristics in both studied groups. All baseline characteristic variables were used for propensity score estimation. The standardized mean difference (SMD) was used as a balance metric to evaluate the difference between distributions of a baseline characteristics variable; moreover, the balance indicator was considered as ‘SMD < 0.1’.

All statistical analyses were conducted applying R version 4.0.3, moreover, we used several packages in R: “survival” (package for survival analysis in R), “survminer” (drawing survival curves), “MatchIt” (propensity score matching), and “ggplot2”. All P-values are two-sided, moreover, P-values < 0.05 were considered statistically significant. The Restricted Cubic Splines (RCS) in the Cox model allows a nonlinear relationship of LDL-C with the “ln hazard ratio” of mortality and MACCE, estimated from the Cox regression model. We considered four knots at the5th, 25th, 75th, and 95th percentile at spline terms.

## Results

### Demographics

In this study, we enrolled 10,218 patients who underwent isolated CABG with preoperative LDL-C ≤ 100 mg/dl. These patients had a mean age of 66.17 ± 9.98 and 2506 (24.5%) of them were women and 7712 (75.5%) were men. The median follow-up was 73.33 [72.15, 74.51] months. Regarding past medical history, 5546 (54.2%) had HTN, 5348 (52.3%) had DLP, 4155 (40.6%) had DM, 358 (3.5%) had the obstructive chronic pulmonary disease (COPD), 277 (2.7%) had a history of heart failure (HF), 250 (2.4%) had a history of renal failure, 3484 (34.0%) have smoked cigarette, and 1492 (14.6) had a history of opium consumption. We divided patients based on their preoperative LDL-C into very low LDL-C (LDL-C ≤ 70 mg/dl) and low LDL-C (70 mg/dl < LDL-C ≤ 100 mg/dl) groups. A detailed comparison of baseline characteristics is demonstrated in Table 1. 5946 (58%) patients were included in the low LDL-C group with a mean age of 66.2 ± 9.8 and consisting of 1614 (27.1%) women and 4332 (72.9%) men. The very low LDL-C group included 4272 (42%) patients with a mean age of 66.2 ± 10.13 whom 892 (20.9%) were women. Number of obese patients (BMI > 30) were significantly higher in low LDL-C group (24.2% vs. 21.4%; P-value = 0.001). DM (P-value = < 0.001) and renal failure (P-value = 0.002) were significantly higher in the very low LDL-C group, while DLP (P-value = 0.002) and COPD (P-value = 0.010) tend to be higher in low LDL-C group (Table 1). After PS matching there were no significant differences between the two studied groups. (Sup Figs. 1 & 2)

**Table 1:** Patients baseline characteristics

		Baseline characteristics of patients before PS matching			Baseline characteristics of patients after PS matching		
Baseline characteristics comparisons		LDL ≤ 70 (4272)	70 < LDL ≤ 100 (5946)	P value	LDL ≤ 70 (3948)	70 < LDL ≤ 100 (3948)	P value
Sex	Female	892 (20.9%)	1614 (27.1%)	< 0.001	841 (21.30%)	854 (21.63%)	0.722
	Male	3380 (79.1%)	4332 (72.9%)		3107 (78.70%)	3094 (78.37%)	
Age		66.18 ± 10.13	66.16 ± 9.84	0.914	66.22 ± 10.15	66.32 ± 9.86	0.650
BMI > 30		907 (21.4%)	1426 (24.2%)	0.001	866 (21.94%)	846 (21.43%)	0.640
**Comorbidities**							
Diabetes		1870 (43.8%)	2285 (38.4%)	< 0.001	1659 (42.0%)	1643 (41.6%)	0.715
Hypertension		2352 (55.1%)	3194 (53.7%)	0.180	2134 (54.1%)	2152 (54.5%)	0.648
Dyslipidemia		2158 (50.5%)	3190 (53.6%)	0.002	1998 (50.6%)	2012 (50.9%)	0.753
Renal failure		129 (3.0%)	121 (2.0%)	0.002	85 (2.2%)	95 (2.40%)	0.451
COPD		126 (3.0%)	232 (3.9%)	0.010	119 (3.0%)	125 (3.2%)	0.696
Family history		1514 (35.4%)	2171 (36.5%)	0.261	1410 (35.7%)	1372 (34.8%)	0.371
**Habitual**							
Current Cigarette smoking		689 (16.2%)	1037 (17.5%)	< 0.001	657 (16.6%)	663 (16.8%)	0.856
Current Opium Consumption		519 (12.2%)	685 (11.6%)	0.247	480 (12.1%)	470 (11.9%)	0.729
**Procedural characteristics**							
Status of procedure	Elective	4125 (96.6%)	5687 (95.8%)	0.026	3817 (96.7%)	3822 (96.8%)	0.759
	urgent or Emergent	143 (3.4%)	250 (4.2%)		131 (3.3%)	126 (3.2%)	
Pre-surgery EF		47.75 ± 9.04	47.62 ± 8.96	0.477	47.78 ± 9.01	47.66 ± 8.91	0.584
Off pump surgery		409 (9.7%)	527 (9.0%)	0.239	362 (9.2%)	380 (9.6%)	0.488
Graft number		3 [3,4]	3 [3,4]	0.964	3 [3,4]	3 [3,4]	0.477
**Medications used**							
ACE Inhibitor or ARB		2908 (68.1%)	3163 (53.2%)	< 0.001	2140 (54.2%)	2394 (60.1%)	0.041
Aspirin		2428 (74.6%)	3479 (73.7%)	0.363	2267 (57.4%)	2344 (59.4%)	0.269
Beta Blocker		2673 (76.8%)	3930 (78.8%)	0.031	2483 (62.9%)	2646 (67.0%)	0.058
Statin		3071 (71.9%)	4312 (72.5%)	0.481	2861 (72.5%)	2891 (72.6%)	0.448
**Lipid profile**							
HDL		36 [36, 37]	37 [37, 38]	< 0.001	37 [36, 38]	37 [36, 38]	0.348

BMI: body mass index; COPD: chronic obstructive pulmonary disease, ACE: angiotensin-converting enzyme, AT1: angiotensin II type 1, EF: ejection fraction

### Primary outcomes

**Fig. 2:**
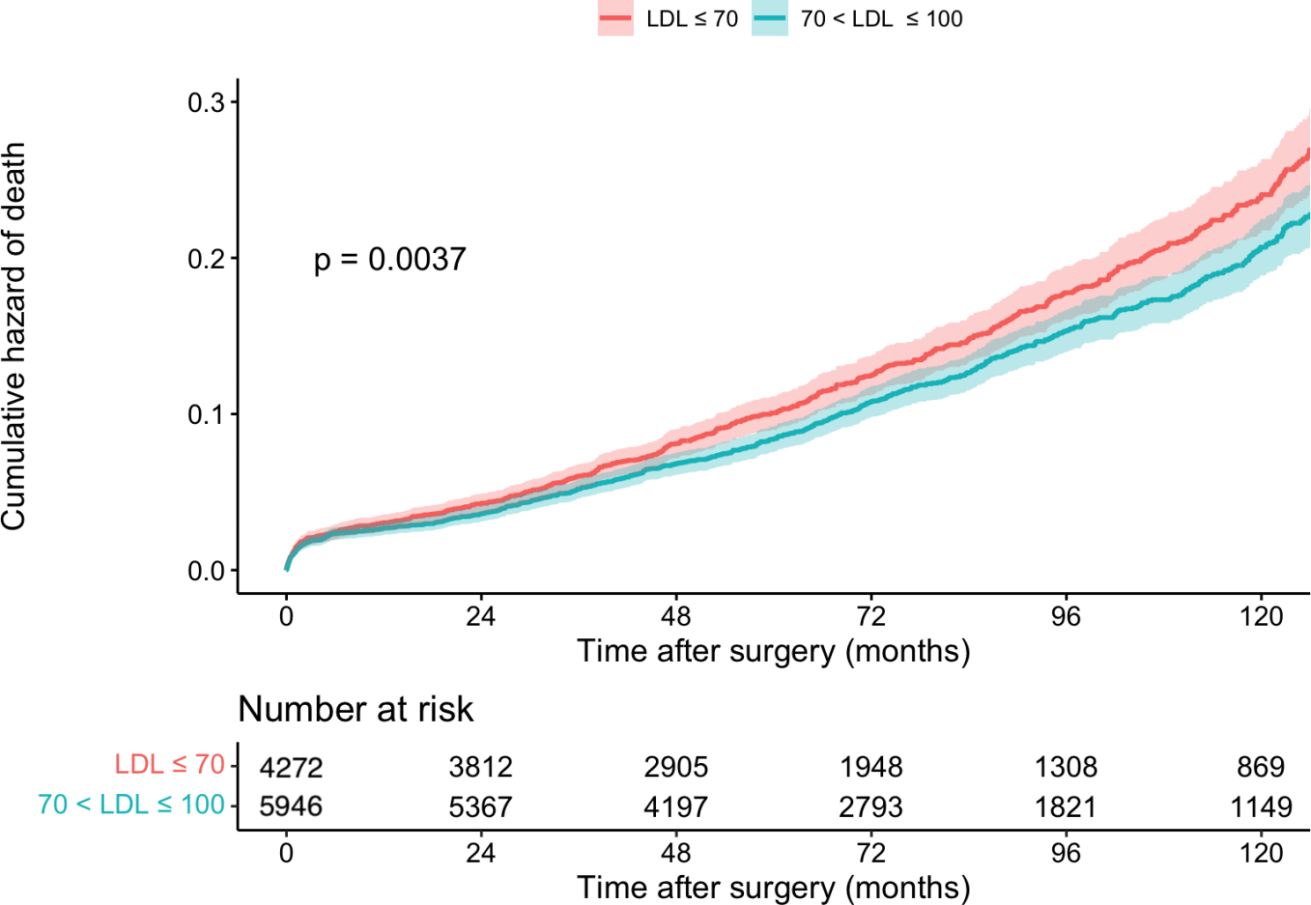
all-cause mortality after CABG in patients with low/very low LDL, before match (n = 7759)

**Fig. 3:**
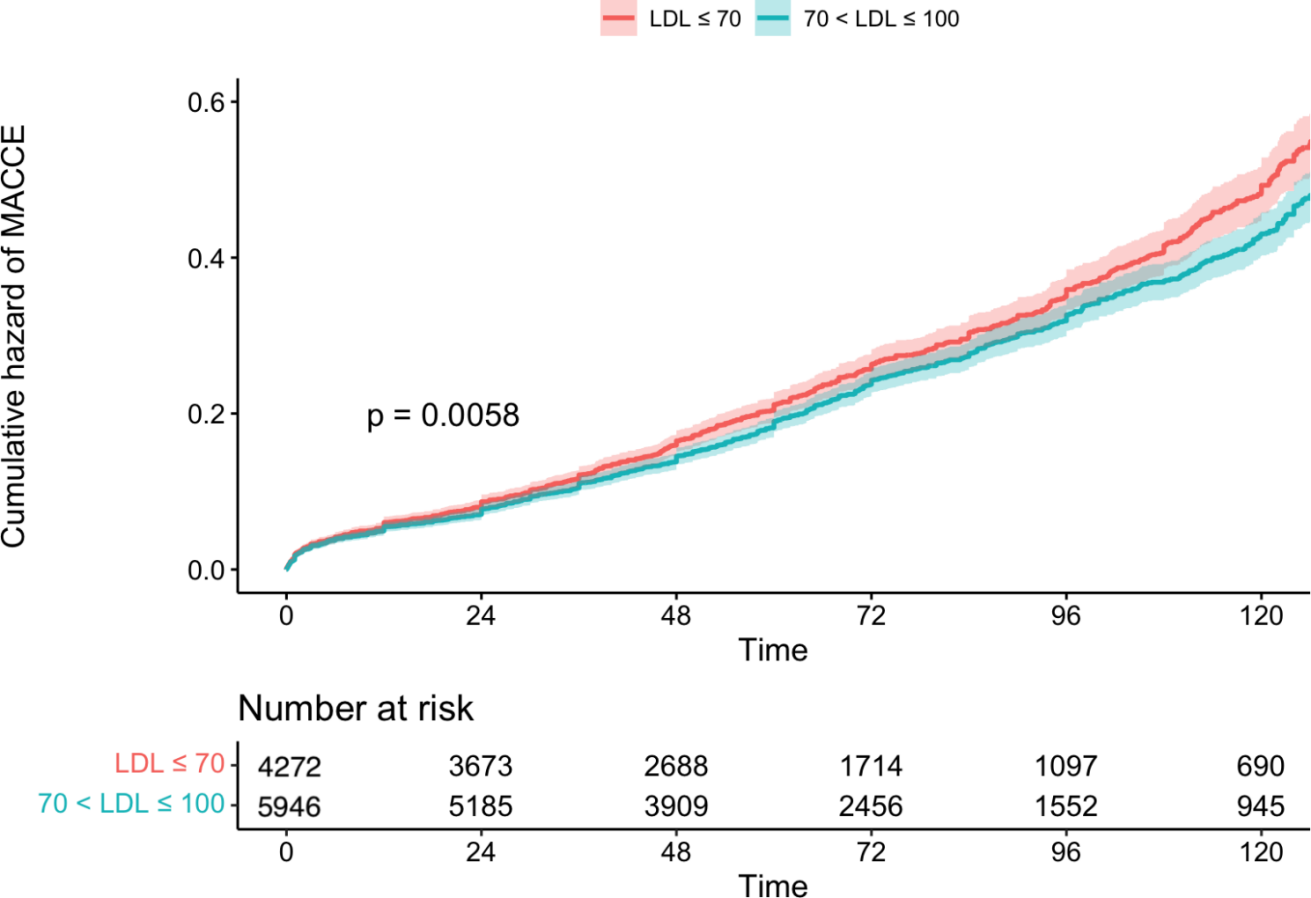
MACCE after CABG in patients with low/very low LDL, before match (n = 7759)

**Fig. 4:**
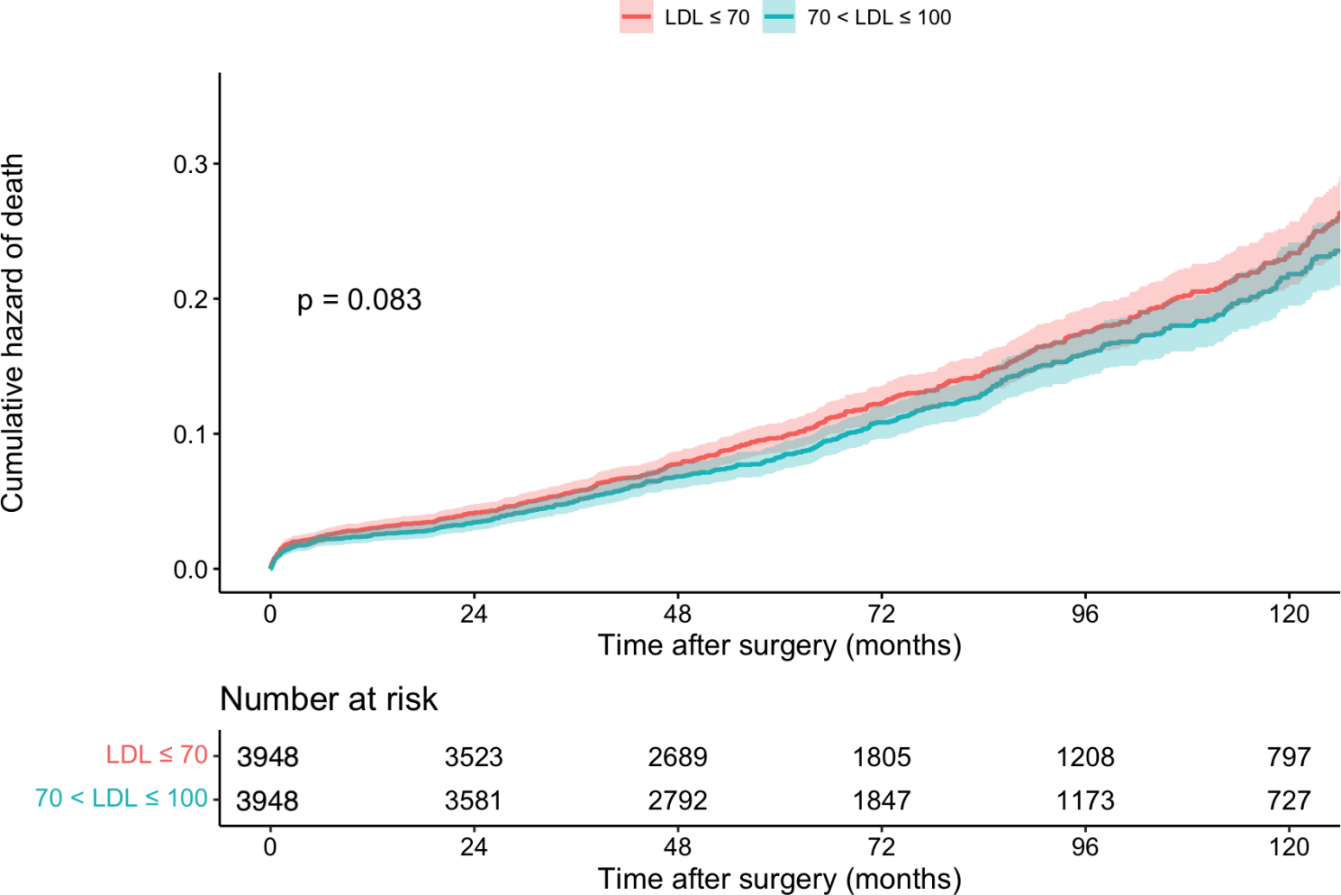
all-cause mortality after CABG in patients with low/very low LDL, after PSM model (n = 7896)

**Fig. 5:**
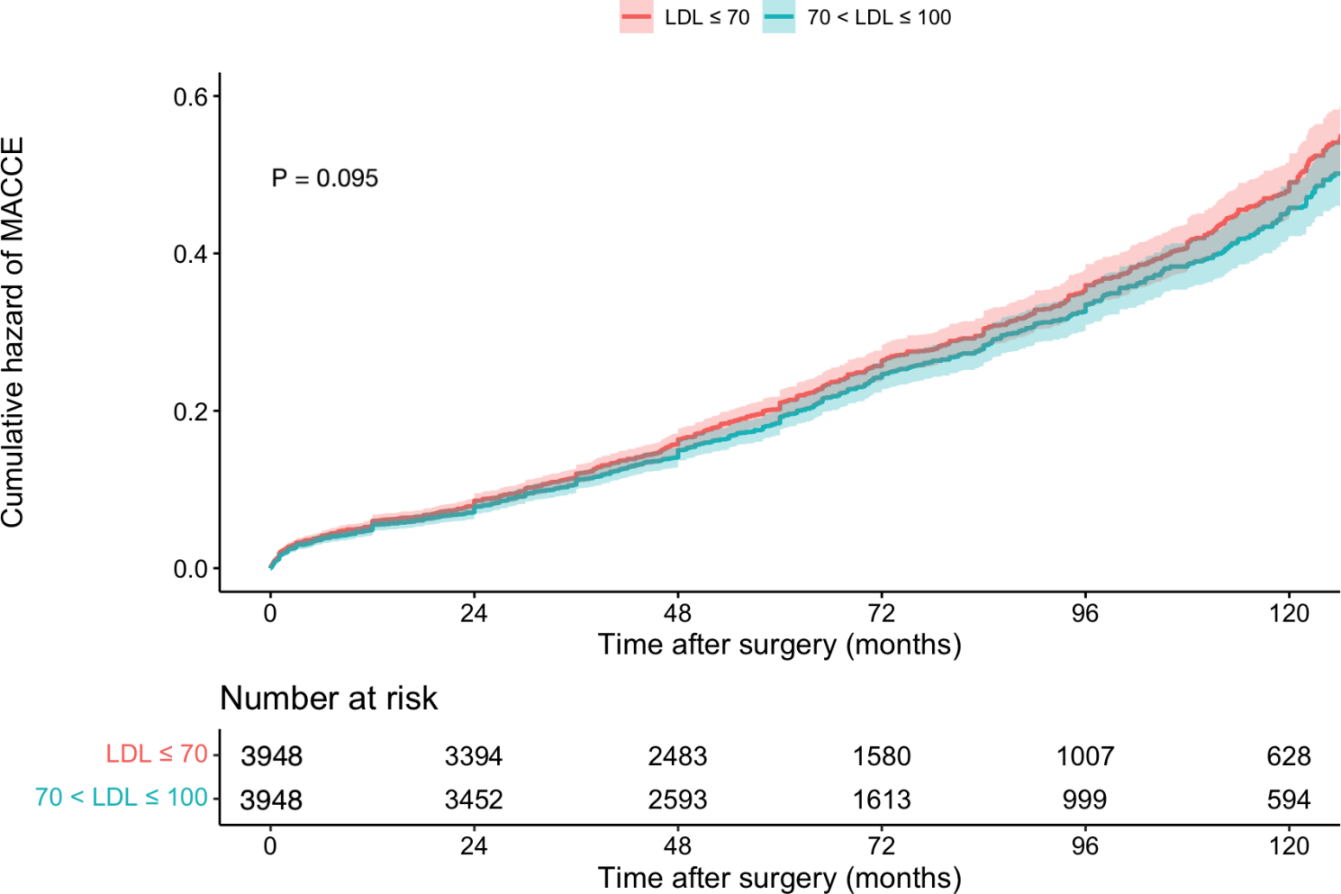
MACCE after CABG in patients with low/very low LDL, after PSM model (n = 7896)

**Fig. 6:**
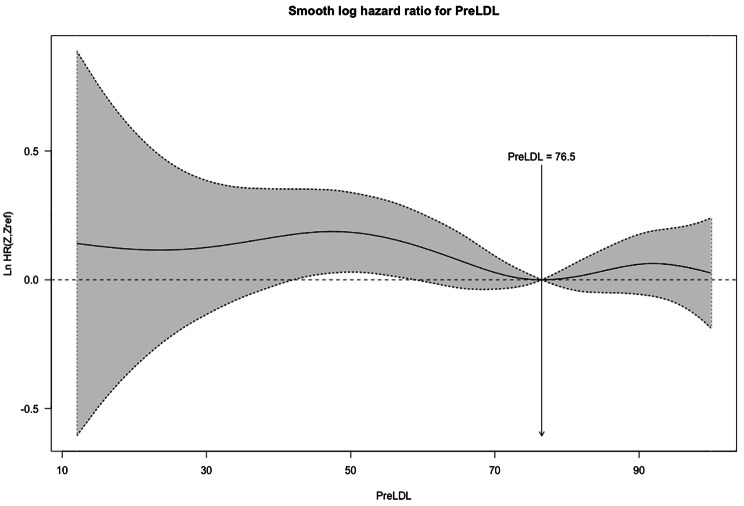
RCS graph for relationship between baseline LDL-C and all-cause mortality

**Fig. 7:**
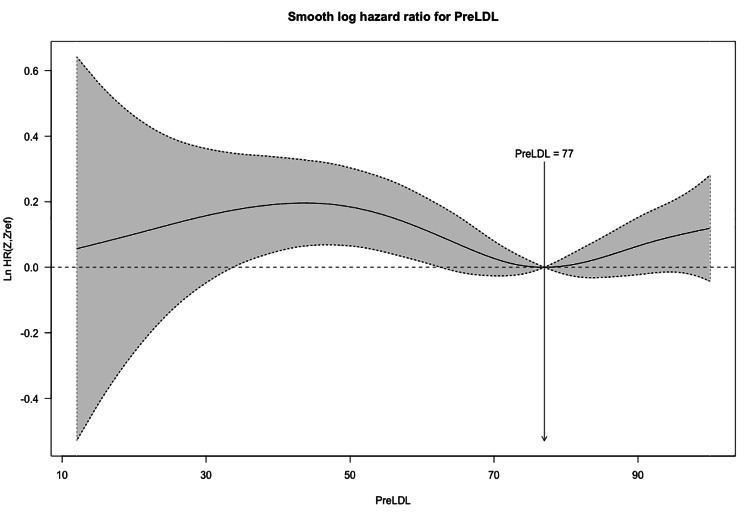
RCS graph for relationship between baseline LDL-C and MACCE

We used Cox regression to investigate LDL-C ≤ 70 effects on all-cause mortality and MACCE in different subgroups. A detailed explanation of the unmatched and matched model used for Cox regression and its result can be seen in Table 2. In the unmatched group, very low LDL-C was associated with significantly higher mortality (14.2% vs. 11.9%, P = 0.004) (Fig. 2) and MACCE (26.0% vs. 23.6%, P = 0.006) (Fig. 3). However, these results were no longer significant after applying PSM model (all-cause mortality HR: 1.115 [95% CI: 0.986–1.262], P = 0.083 (Fig. 4) and MACCE HR: 1.077 [95%CI: 0.984–1.177], P = 0.095 (Fig. 5)). We also performed a sensitivity analysis to eliminate the statin effect on survival in an adjusted model including only patients taking statins. In both the unmatched and PSM models, patients in the very low LDL-C group tend to have higher mortality (Unmatched model HR: 1.239 [95% CI:1.079–1.423; P-value = 0.002]) and MACCE (Unmatched model HR: 1.124 [95% CI:1.017–1.242; P-value = 0.022]). After applying the PSM model, all-cause mortality was significantly higher (HR: 1.207 [95% CI: 1.042–1.336], P = 0.018) but MACCE was no longer significantly higher in the very low LDL-C group (HR: 1.114 [95% CI: 0.996–1.246], P = 0.065) compared to low LDL-C group (Table 3). In addition, the RCS graphs demonstrated no monotonic positive linear relationship between baseline LDL-C and primary outcomes; patients with baseline LDL-C ≈ 77 had the lowest risk of all-cause mortality (Fig. 6) and MACCE (Fig. 7). Moreover, a U-shaped association between baseline LDL-C level and primary outcomes was observed in studied patients (Figs. 6 and 7).

**Table 2:** Association of primary outcomes with LDL-C ≤ 70 vs. 70 < LDL-C < 100 (n = 10,218)

	No (LDL ≤ 70/ 70 < LDL ≤ 100)	Crude model		PSM model	
		HR (95% CI)	P value	HR (95% CI)	P value
All-cause mortality	593 (14.2%)/ 697 (11.9%)	1.175 (1.054–1.310) *	0.004	1.115 (0.986–1.262)	0.083
MACCE	1086 (26.0%)/ 1378 (23.6%)	1.118 (1.033–1.211) *	0.006	1.077 (0.984–1.177)	0.095

MACCE: major adverse cardiac and cerebrovascular events, HR: hazard ratio, CI: confidence interval

* P-value < 0.05

**Table 3:** Sensitivity analysis for the association of primary outcomes whit LDL-C ≤ 70 vs. 70 < LDL-C < 100 in statin user patients (n = 7,383)

	No (LDL ≤ 70/ 70 < LDL ≤ 100)		Crude model		PSM model	
	Statin Users (n = 7,383)	Statin non-users (n = 2,835)	HR (95% CI)	P value	HR (95% CI)	P value
All-cause mortality	369 (12.1%)/ 441 (10.2%)	224 (18.1%)/ 256 (16.1%)	1.239 (1.079–1.423)	0.002	1.207 (1.042–1.336)	0.018
MACCE	676 (22.1%)/ 900 (20.8%)	410 (33.1%)/ 478 (30.0%)	1.124 (1.017–1.242)	0.022	1.114 (0.996–1.246)	0.065

MACCE: major adverse cardiac and cerebrovascular events, HR: hazard ratio, CI: confidence interval

### Secondary outcomes

Regarding secondary outcomes during our follow-up, there was no significant difference between the two groups in CVEs, however patients with LDL-C ≤ 70 mg/dl were more likely to have a cerebrovascular accident (CVA) as a part of CVE (HR: 1.343 [95% CI: 1.053–1.714;

P-value = 0.003]). We also compared non-fatal acute coronary syndrome (ACS), in-hospital mortality, intubation time, and intensive care unit (ICU) length of stay which showed no significant difference between the two groups (Table 4).

**Table 4:** Association of Secondary outcomes with LDL-C ≤ 70 vs. 70 < LDL-C < 100

Outcomes		Crude model			PS matched		
		No (LDL ≤ 70/ 70 < LDL ≤ 100)	HR, OR	P value	No (LDL ≤ 70/ 70 < LDL ≤ 100)	HR, OR	P value
Non-fatal CVEs		493 (11.8%)/ 681 (11.7%)	1.026 (0.913–1.152) *	0.872	465 (12.0%)/ 465 (12.0%)	1.032 (0.891–1.195)	0.674
	ACS	367 (8.8%)/ 547 (9.4%)	0.950 (0.832–1.085) *	0.231	349 (9.0%)/ 364 (9.4%)	0.862 (0.660–1.125)	0.275
	CVA	126 (3.0%)/ 133 (2.3%)	1.343 (1.053–1.714) *	0.003	116 (3.0%)/ 101 (2.6%)	1.145 (1.012–1.297)	0.033
Revascularization		0 / 1	-	-	-	-	-
In hospital mortality		63 (1.5%)/ 77 (1.3%)	1.147 (0.820–1.603) #	0.112	57 (1.5%)/ 46 (1.2%)	1.234 (0.894–1.856)	0.261
Intubation time (hour)		14.701 ± 9.012/ 14.626 ± 10.213	-	0.818	14.55 ± 9.012/ 14.50 ± 10.318	-	0.965
Length of ICU admission (hour)		50.691 ± 12.093/ 51.054 ± 12.563	-	0.943	51.234 ± 12.123/ 51.454 ± 12.163	-	0.832

CVE: cardiovascular event, ACS: acute coronary syndrome, CVA: cerebrovascular accident, HR: hazard ratio, OR: odds ratio, ICU: intensive care unit

* HR, # OR

## Discussion

The results of the present prospective registry-based study with a large sample size demonstrated a significant association between very low perioperative LDL-C levels (LDL-C ≤ 70 mg/dl) and higher all-cause mortality and MACCE, compared to low LDL-C levels (70 mg/dl < LDL-C ≤ 100 mg/dl) in patients who underwent isolated CABG in unmatched groups. Whilst, after applying the PSM model for confounding variables, there was no significant difference between low and very low LDL-C in primary outcomes. In addition, the sensitivity analysis to remove the effect of statin consumption has suggested patients with the very low LDL-C group have higher mortality in both unmatched and the PSM model, but MACCE was no longer significantly higher in the very low LDL-C group after applying the PSM model. In addition, no monotonic positive linear correlation between baseline LDL-C and primary outcomes was found, with baseline LDL-C ≈ 77 being associated with the lowest risk of MACCE and mortality after CABG.

Most previous epidemiologic surveys have indicated that elevated LDL-C levels increase the risk of atherosclerosis and cardiovascular events and LDL-C reduction is considered a principal goal for primary and secondary prevention of cardiovascular disease, particularly for patients who have undergone CABG [6, 8, 20, 21]. While several previous studies had reported that baseline hypercholesterolemia was associated with a lower risk of adverse outcomes, Wang et al. reported that this correlation was not observed among NSTEMI patients with undiagnosed hypercholesterolemia, suggesting that the lower risk appreciated in patients with hypercholesterolemia, the so-called cholesterol paradox, may have been due to a higher likelihood of prior medical contact and consequent statin therapy [22]. Furthermore, according to several studies, a negative correlation between baseline or admission serum LDL-C levels and clinical outcomes exist in certain subgroups of patients with coronary artery disease. In the line of our study, a recent observation showed that a very low baseline LDL-C level (< 70 mg/dL) conferred a worse prognosis among patients with CAD and advanced kidney disease [12]. Consistently, Mallah et al., found that low serum LDL-C (≤ 105 mg/dl) at admission was associated with higher long-term all-cause mortality in NSTEMI patients [11]. Reddy et al.’s findings also showed that in AMI patients, lower baseline serum LDL-C levels (< 77 mg/dl) were associated with an increased risk of in-hospital mortality in acute phases [23]. Similarly, Nakahashi et al. concluded that ACS patients with low serum LDL-C concentrations on admission were more likely to experience adverse outcomes [24]. On the other hand, there are reports that a very low cholesterol level is associated with a poorer prognosis in patients with established heart failure [25, 26].

[27][28, 29][30] Several hypotheses could be construed based on this finding. First, according to our results, patients with very low LDL-C concentrations (< 70 mg/dl) experienced a higher prevalence of prognostically-relevant cardiovascular disease-related comorbidities, including diabetes mellitus and renal failure. Similarly, an intracoronary imaging study suggested that ACS patients with decreased LDL-C on admission may have a greater number of residual risk factors [31]. Moreover, findings of a recent investigation showed that patients with LDL-C < 70 had higher prevalence of diabetes mellitus, hypertension, anemia, and poor renal function among patients [12]. In this regard, the results of the propensity score matching analysis did not elucidate a significant difference between low and very low LDL-C groups in the all-cause mortality and MACCE, which in turn reflects that prognosis of the individuals with very low LDL-C might be worse due to higher prevalence of comorbidities. Taken together, it seems important to consider the concomitant conditions rather than the net LDL-C concentration for risk assessment.

A second explanation may be that patients with higher cholesterol concentrations are more likely to be taking medications such as β-blockers and angiotensin-converting enzyme inhibitors before their admission; as a result, these medications may individually or synergistically contribute to the paradoxically lower risk of patients with 70 mg/dl < LDL-C ≤ 100 mg/dl as compared to patients with LDL-C < 70 mg/dl. A third explanation may be that in patients with very low serum LDL-C levels, HDL-C levels are also comparatively lower, which may be prognostically more impactful than the former [32]. Accordingly, Ogita et al. revealed that, despite achieving optimal LDL-C control after coronary revascularization, diabetic patients with low serum HDL-C levels had higher rates of major cardiovascular events [33]. Moreover, it has been suggested in another study that low serum HDL-C level might be an independent prognostic factor for significant atherosclerotic progression post-CABG [34]. Results of a recent meta-analysis suggested that lower serum HDL-C was correlated with an increased risk of MACCE, all-cause mortality, and cardiac death, whereas serum LDL-C level was not associated with these outcomes in coronary heart disease patients [35]. However, PSM analysis for the above confounding variables demonstrated no association between serum LDL-C ≤ 70 mg/dL and higher all-cause mortality and worse long-term outcomes in CABG patients.

A recent study demonstrated that patients with very low baseline serum LDL-C (< 70 mg/dl) were more likely to have underlying malnutrition; the worse long-term prognosis seen in these patients may be mediated in part by the higher prevalence of malnutrition potential side effects in this patient group. Moreover, after adjusting for the effect of malnutrition, this cholesterol paradox disappeared [36].

It is indicated that perioperative statin therapy in patients undergoing isolated CABG was associated with significant and dose-dependent improvement in early prognosis [27]. The most striking benefits of LDL-C lowering therapy were observed among patients with elevated cholesterol levels. Although this effect has been seen to a lesser extent in the majority of patients with CAD who have average cholesterol levels, this may not be generalized to populations with lower baseline LDL-C concentrations [28, 29]. Besides, it has been reported that the amount of LDL-C lowering may not predict the beneficial effects of statin therapy on cardiovascular disease outcomes [30]. A recent meta-analysis demonstrated that LDL-C lowering therapy was of no benefit to patients with baseline serum LDL-C levels of less than 100 mg/dL, despite being significantly beneficial in patients with higher baseline LDL-C levels [28]. This study was not intended to evaluate the impact of treatment interventions on lipid levels and outcomes, and as such, this analysis was adjusted for preadmission statin treatments. Therefore, this article does not stand against statin therapy in patients. Instead, we evaluated the outcomes, regardless of statin consumption, among patients with LDL-C ≤ 70 mg/dl and 70 mg/dl < LDL-C ≤ 100 mg/dl.With respect to the association between very low serum LDL-C levels (≤ 70 mg/dl) and increased long-term all-cause mortality in patients who have undergone isolated CABG, our data may be able to provide new insight into the clinical management of serum LDL-C in this population. Excessively low baseline serum LDL-C levels may occur as a result of multiple disease processes, adverse conditions, and possible genetic vulnerabilities; therefore, a careful assessment and, if necessary, screening for possible comorbidities should be performed. In addition to the baseline serum LDL-C level, careful attention must also be paid to the baseline serum HDL-C level, because of its prognostic significance. Ultimately, further multicenter studies should be performed to accurately determine the relationship between baseline serum LDL-C, confounding factors, and patient outcomes in order to better guide lipid level and risk factor management.

### Limitations

In this study, we did not aim to compare the low/very low LDL group with patients with higher LDL-C concentrations, which may be considered a limitation or strength. Based on guidelines, secondary prevention with high-dose statin will be initiated after CABG and before discharge regardless of baseline LDL-C level. Patients with high LDL-C levels may benefit more from high-dose statin therapy during follow-up. Hence the paradoxical effect of baseline LDL-C will be exaggerated if we include these patients. In addition, since assessing statin therapy during follow-up (in terms of compliance, dosage, and duration) was beyond the scope of the present study, patients with higher LDL-C levels were not included. Also, we were aware of the beneficial effects of LDL-C lowering therapy, in terms of LDL-C levels < 55 mg/dl, but in the current study, we focused on the impact of baseline preoperative LDL-C levels on the outcomes after CABG and did not assess the benefit of LDL-C lowering therapy during follow-up periods.

Besides, evaluating and comparing the goal of LDL-C treatment after CABG was beyond the scope of the present study. We excluded patients with a previous history of cardiovascular disease, but we cannot consider our patients a homogenous population. In our registry protocol, we evaluated the patients in terms of their risk factors and treatment yearly, however, since no assessments were conducted during one year, this could be considered a limitation of our study.

## Conclusion

In conclusion, it seems that very low serum LDL-C levels (≤ 70 mg/dl) could be associated with increased long-term all-cause mortality and cardiovascular events in patients who have undergone isolated CABG. However, these results should be evaluated with caution. Our data may be able to provide an insight regarding the clinical management of serum LDL-C in this population. It should not be implied that patients with a very low LDL-C have a necessarily better outcome after CABG. Individualized risk stratification should be done regardless of baseline LDL-C.

Ultimately, further multicenter studies should be performed to accurately determine the relationship between baseline serum LDL-C, confounding factors, and patient outcomes to improve CAD risk factor management.

## Electronic supplementary material

Below is the link to the electronic supplementary material.

**Additional File 1:** Frequency of Propensity Scores in each LDL group before and after matching

## Data Availability

The data that support the findings of this study are available on request from the corresponding author (K.H).

## List of abbreviations

ACS: acute coronary syndrome
CABG: coronary artery bypass grafting
CAD: coronary artery disease
COPD: obstructive chronic pulmonary disease
CS: cigarette smoking
CVA: cerebrovascular accident
CVEs: cardiovascular events
DLP: dyslipidemia
DM: diabetes mellitus
HbA1c: hemoglobin A1c
HDL-C: high-density lipoprotein cholesterol
HF: heart failure
HR: hazards ratio
HTN: hypertension
ICU: intensive care unit
LDL-C: low-density lipoprotein-cholesterol
MACCE: major adverse cardio-cerebrovascular events
NSTEMI: non-ST elevation myocardial infarction
PSM: propensity score matching
