# Supplementary material for: The prognostic role of the low and very low baseline LDL-C level in outcomes of patients with cardiac revascularization; comparative registry-based cohort design
Source: J Cardiothorac Surg. 2023 Jul 28;18:240. doi: 10.1186/s13019-023-02333-y (PMC10386279; doi:10.1186/s13019-023-02333-y)
Supplement: Supplementary file 1 — Additional File 1: Frequency of Propensity Scores in each LDL group before and after matching [file 13019_2023_2333_MOESM1_ESM.docx]

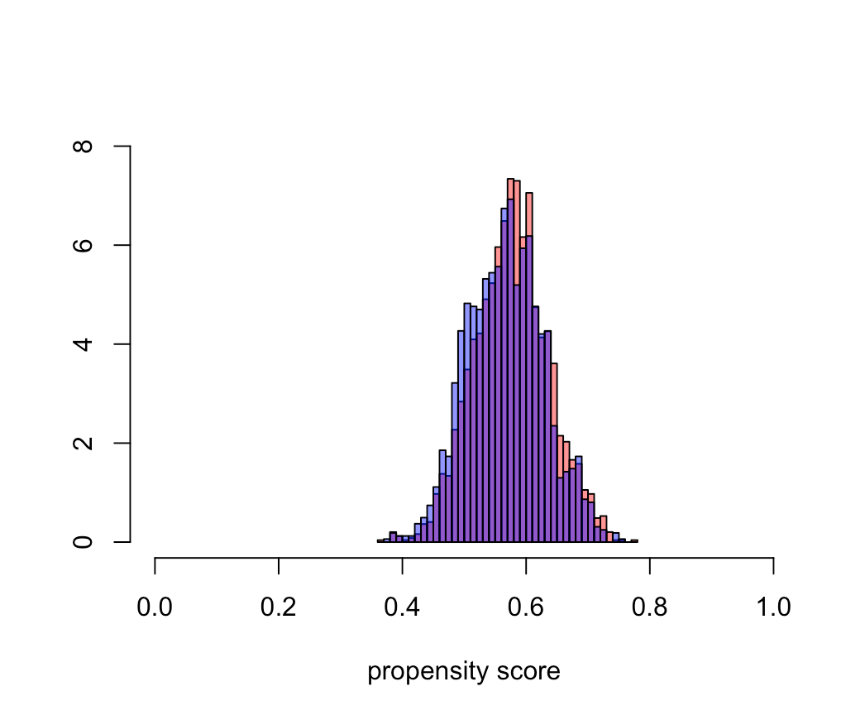


Sup Figure 1: Frequency of Propensity Scores in each LDL group before matching. Blue (70 > LDL), Red (70 < LDL < 100)


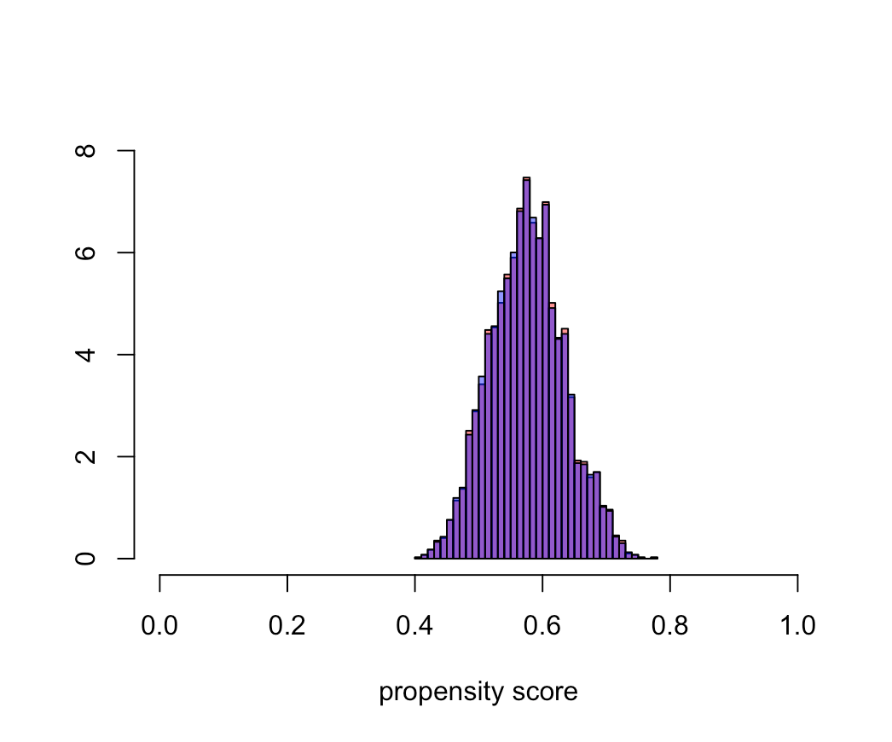


Sup Figure 2: Frequency of Propensity Scores in each LDL group after matching. Blue (70 > LDL), Red (70 < LDL < 100)
